# Five-Year Outcomes of Hybrid Arch Frozen Elephant Trunk Repair With Novel Multibranched Hybrid Graft

**DOI:** 10.1016/j.atssr.2023.06.009

**Published:** 2023-07-14

**Authors:** Junichi Shimamura, Rami Abazid, Jill Gelinas, Matthew Valdis, Audra Duncan, Adam Power, Luc Dubois, Michael W.A. Chu

**Affiliations:** 1 Division of Cardiac Surgery, Western University, London Health Sciences Centre, London, Ontario, Canada; 2 Division of Cardiology, Western University, London Health Sciences Centre, London, Ontario, Canada; 3 Division of Vascular Surgery, Western University, London Health Sciences Centre, London, Ontario, Canada

## Abstract

**Background:**

The objective of this study was to report the 5-year outcomes of hybrid arch frozen elephant trunk (FET) procedures with a multibranched hybrid graft.

**Methods:**

Between 2014 and 2020, 50 consecutive patients (63 ± 15 years old; 34% women) underwent hybrid arch FET with Thoraflex hybrid graft (Terumo Aortic) at a single center. Indications included aortic aneurysm (n = 48 [96%]), acute aortic dissection (n = 10 [20%]), and chronic dissection (n = 20 [40%]). Follow-up was complete, and mean follow-up was 1455 ± 664 days.

**Results:**

All 50 patients experienced successful device implantation. The 30-day/in-hospital mortality was 2% (n = 1). Stroke and transient neurologic deficits occurred in 1 patient (2%) and 3 patients (6%). Two patients (4%) and 1 patient (2%) experienced transient and permanent spinal cord ischemia. FET thromboembolic complication was observed in 1 patient (2%). In follow-up, 6 patients died of aortic events, and there were 13 reinterventions in the downstream aorta, of which 46% (6/13) were planned second-stage operations. Survival rate at 1 year, 2 years, and 5 years was 96%, 92%, and 85%, and freedom from unplanned distal reintervention at 1 year, 2 years, and 5 years was 98%, 92%, and 81%. Computed tomography follow-up demonstrated positive distal aortic remodeling with aneurysmal regression and stabilized aortic dimensions in patients with aortic dissection.

**Conclusions:**

The hybrid arch FET procedure with a novel hybrid graft is associated with good early and midterm outcomes. Longer term outcomes merit further investigation.

## Visual Abstract

**Figure:**
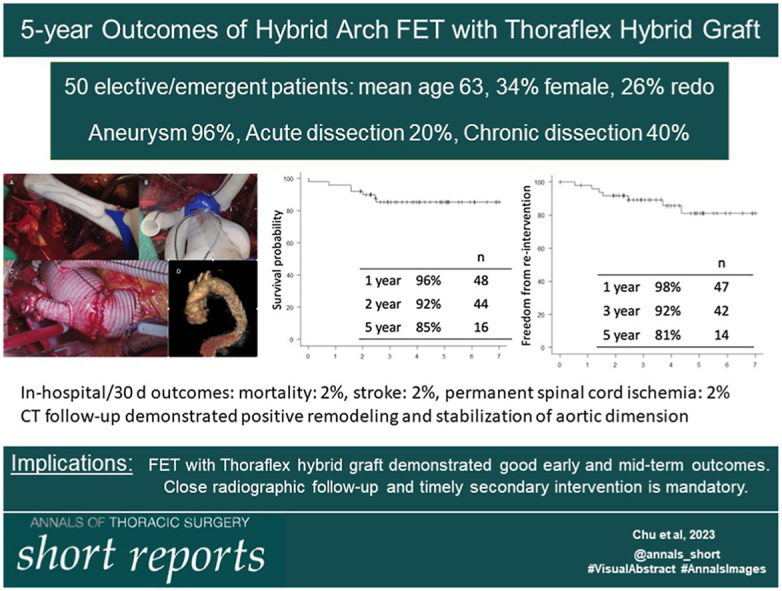

**In Short**

▪ Frozen elephant trunk procedure with Thoraflex hybrid graft is associated with good early and midterm outcomes.
▪ Imaging follow-up demonstrated positive distal aortic remodeling with aneurysmal regression and stabilized aortic dimensions in patients with aortic dissection.
▪ Further experience and longer follow-up of this device will help establish its role in hybrid aortic arch repair for complex aortic disease.

Contemporary advances in aortic arch reconstructive surgery reflect a growing interest in innovative, hybrid arch frozen elephant trunk (FET) repair techniques. In North America, off-label thoracic endovascular aortic repair (TEVAR) procedures have been popularized in aortic arch repair because of availability, ease of use, and lack of hybrid graft alternatives. However, these strategies have been associated with higher risk of stroke, stent migration, and type 1A endoleaks.^1^^,^^2^ Proximal endoleaks remain challenging to treat and in many cases require multiple complex reintervention.

The Thoraflex hybrid graft (Terumo Aortic) has recently received Food and Drug Administration approval for complex aortic arch repair and has the potential to revolutionize the field.^3^, ^4^, ^5^ This hybrid graft enables single-stage repair in selected patients and provides a favorable landing zone for distal extension. Importantly, it has a surgically sewn aortic arch anastomosis that abolishes the risk of type 1A endoleaks. The aim of this study was to report midterm outcomes of FET using this hybrid graft.

## Patients and Methods

In this study, 50 consecutive patients who underwent hybrid arch FET repair with Thoraflex hybrid graft between 2014 and 2020 were analyzed.

### Patient Characteristics

Mean age was 63 ± 15 years, and 17 (34%) patients were female (Supplemental Table). Surgical indication included aortic aneurysm in 48 (96%), chronic aortic dissection in 20 (40%), acute aortic dissection in 10 (20%), and aortic rupture in 4 (8%) patients. Thirteen patients (26%) had undergone previous sternotomy. Regarding the urgency of the operation, elective, urgent, and emergent operation was performed in 27 (54%), 10 (20%), 13 (26%).

### Surgical Technique

Details of surgical technique have been previously reported^6^ and are described in the surgical Video.

### Aortic Measurement and Follow-up

Serial imaging follow-up was performed by computed tomography angiography before discharge, postoperatively at 8 to 12 weeks, and then annually. We used dedicated software (AQi; TeraRecon) and calculated the aortic volume and true and false lumen by segment (ascending aorta, FET level, distal thoracic aorta).

### Statistics

All data were collected retrospectively in a computerized institutional database and imported into JMP version 13.2.0 (SAS Institute) for statistical analysis. Continuous variables are presented as mean ± SD.

## Results

### Operative Details

The Thoraflex device was successfully implanted in all cases (Table 1). The mean cardiopulmonary bypass time was 262 ± 74 minutes; cross-clamp time, 167 ± 70 minutes; and circulatory arrest time, 42 ± 11 minutes. The mean hypothermic arrest temperature was 25 ± 1 °C.

**Table 1:** Operative Characteristics

	No. (%)
Innominate reconstruction	
Anatomic	50 (100)
Carotid reconstruction	
Anatomic	47 (94)
Extra-anatomic	3 (6)
Subclavian reconstruction	
Anatomic	4 (8)
Extra-anatomic	44 (88)
Ligation	2 (4)
Preoperative subclavian transposition	33 (66)
Concomitant procedures	
Ross procedure	1 (2)
Valve-sparing root replacement	6 (12)
Bentall procedure	5 (10)
Aortic valve replacement	9 (18)
Coronary artery bypass grafting	4 (8)
Urgency	
Elective	27 (54)
Urgent	10 (20)
Emergent	13 (26)

### In-Hospital or 30-Day Outcomes

One patient (2%) died suddenly on postoperative day 7 while awaiting hospital discharge (Table 2). The autopsy demonstrated myocardial infarction related to an acute plaque rupture in the distal right coronary artery. One patient (2%) suffered a postoperative stroke, and 3 patients (6%) had temporary neurologic dysfunction. Two patients (4%) experienced transient spinal cord ischemia (SCI) and 1 patient (2%) had permanent SCI (occurring in patients with aortic dissection). FET thrombus was observed in 1 patient (2%).

**Table 2:** In-Hospital/30-Day Complications

	No. (%)
Mortality	1 (2)
Stroke	1 (2)
Temporal neurologic deficit	3 (6)
Delirium	11 (22)
Transient spinal cord injury	2 (4)
Permanent paraplegia	1 (2)
Reoperation for bleeding	3 (6)
Myocardial infarction	1 (2)
Renal failure	3 (6)
Serious arrhythmia	2 (4)
Sepsis	3 (6)
Prolonged mechanical ventilation	10 (20)
Recurrent laryngeal nerve injury	1 (2)
Thromboembolic event	1 (2)

### Survival and Follow-up Outcomes

**Figure 1:**
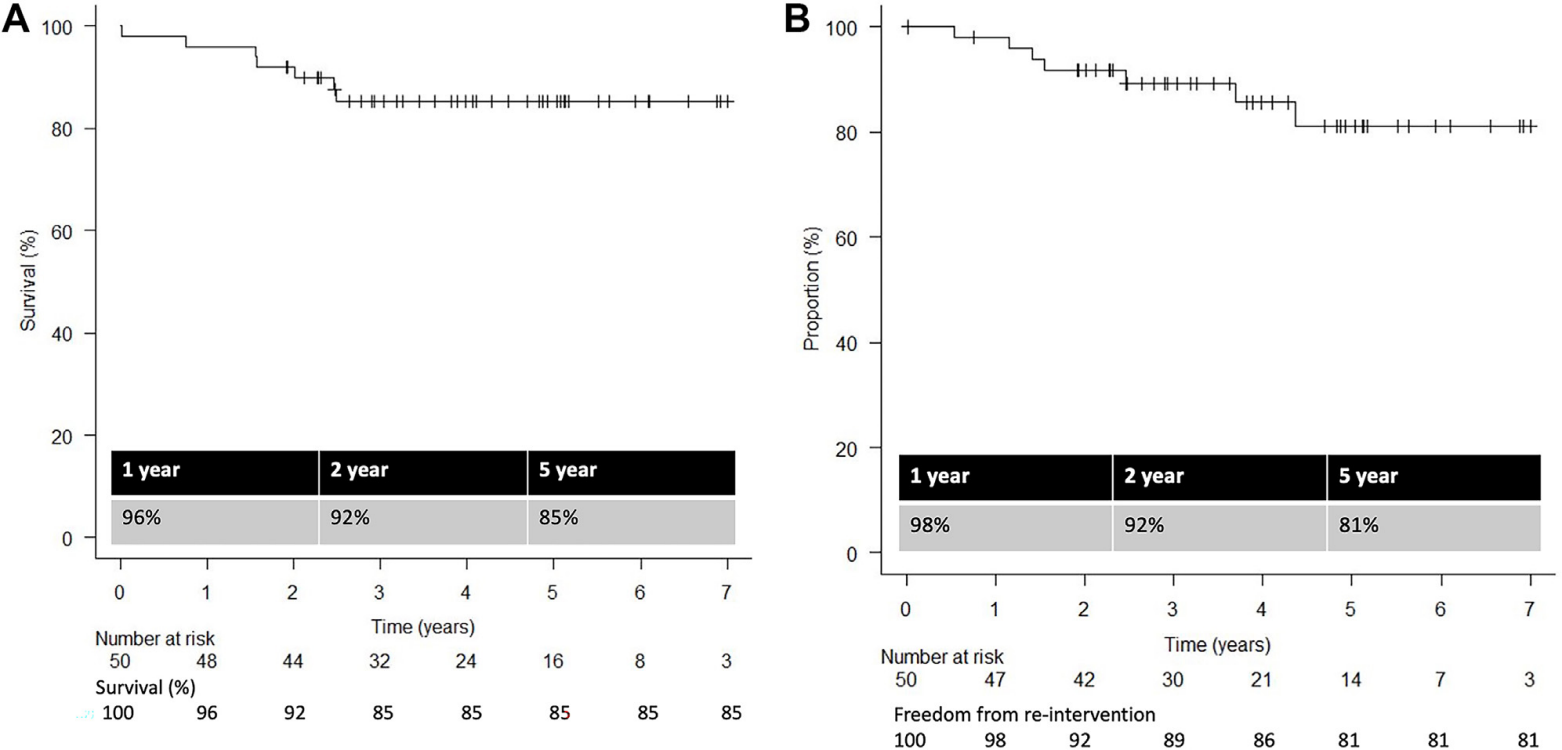
Kaplan-Meier curve of (A) survival and (B) freedom from reintervention.

During a mean follow-up of 1455 ± 664 days, 6 patients died. Causes of death included respiratory failure after the second-stage thoracoabdominal aortic aneurysm repair, sepsis and bleeding complication after the second-stage TEVAR, rupture of the remnant aneurysm in 2 patients awaiting intervention, and graft infection in 1 patient. There were 13 reintervention procedures in the downstream aorta (open surgery [n = 1] or endovascular [n = 12]), and 46.2% (6/13) were planned second-stage operations. Unplanned reintervention was performed in patients with chronic dissection (n = 4) and aneurysm (n = 3). Survival rates at 1 year, 2 years, and 5 years are 96%, 92%, and 85% (Figure 1A). Freedom from unplanned distal reintervention at 1 year, 2 years, and 5 years is 98%, 92%, and 81% (Figure 1B).

### Imaging Follow-up

**Figure 2:**
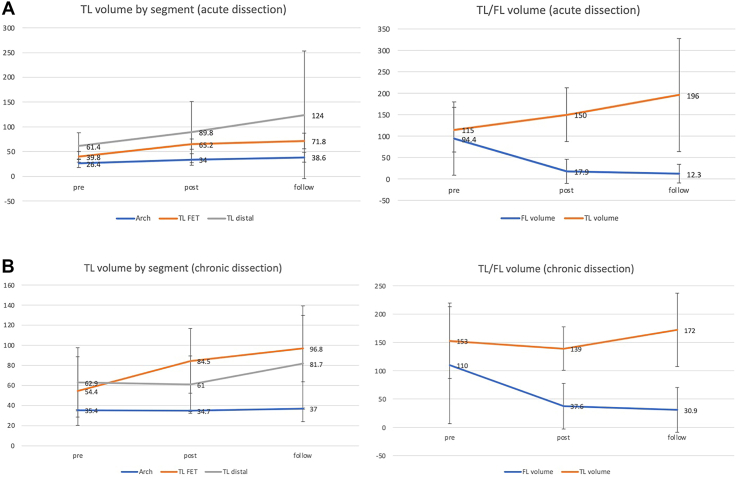
Volumetric assessment of cases with (A) acute dissection and (B) chronic dissection. (FET, frozen elephant trunk; FL, false lumen; TL, true lumen.)

In patients with acute and chronic aortic dissection, FET implantation expanded the true lumen and remodeled the false lumen during the follow-up (Figure 2). Type 1B (unanticipated), type 2, and type 3 endoleaks were observed in 1 patient, 2 patients, and 1 patient, respectively, but type 1A endoleak was not observed.

## Comment

In this study, we describe 5-year outcomes of the FET procedure using a multibranched hybrid graft for a variety of aortic arch disease and demonstrate a low mortality and an acceptable distal reintervention rate. We believe that this approach is straightforward and reproducible and provides an innovative solution to address some of the challenges with off-label endovascular stent graft use in complex arch surgery.

We previously reported the short-term results of FET from 9 Canadian centers,^6^ and in-hospital mortality, stroke, transient SCI, and permanent SCI rates were 5%, 5%, 5%, and 0%. The survival rate at 1 year and 2 years was 95% and 90%. These results are comparable to the recent 1-year US multicenter investigational device exemption trial, which reported that the mortality, stroke, and permanent paraplegia rates were 11%, 5%, and 5%.^4^ Although this represented our inaugural experience, we demonstrated good early results of in-hospital mortality, stroke, and permanent paraplegia rates of 2%, 2%, and 2%. Our study also added evidence on the midterm outcomes with the survival rate at 1 year, 2 years, and 5 years of 96%, 92%, and 85%. This is in keeping with results from European and Asian aortic centers. Shrestha and coworkers^7^ reported a survival rate of 81% for 100 patients during follow-up at 3.1 ± 1.4 years. They reported that 22% of patients received treatment of the downstream aorta, with most (68%) being anticipated second procedures. Katayama and colleagues^8^ observed 120 patients with type A aortic dissection for 15 years, and 5 patients (4.2%) required distal reoperations. In our series, remote death occurred in 6 patients; 3 occurred after second-stage interventions, 2 were remnant aneurysm ruptures, and 1 was a FET infection. Therefore, close imaging follow-up of remnant aorta and timely reintervention are mandatory. Regarding the morphologic changes, Shrestha and coworkers^7^ performed a radiologic follow-up based on cause. They reported that FET implantation stabilized the aorta at FET level in patients with aneurysms. For patients with aortic dissection, FET implantation resulted in increase of true lumen diameter in the stented segment with a positive aortic remodeling. Our results are consistent with these findings.

We believe the FET technique continues to gain popularity because of the following potential benefits^5^^,^^9^:

• FET enables single-stage repair in selected patients.
• FET improves distal malperfusion and promotes favorable distal aortic remodeling in patients with acute aortic dissection.
• FET provides an attractive landing zone for distal reintervention for TEVAR extension.

There is no doubt that early in our series, patients were highly selected; however, with time, we have expanded indications to include most patients presenting with acute aortic syndromes and complex arch anatomy. We think that the learning curve is relatively short for aortic surgeons, and the operation is relatively straightforward and reproducible; the good early results have now been demonstrated to be generalizable. In Canada, we have been fortunate to have access to multiple FET graft options but have become comfortable with the Thoraflex hybrid prosthesis because of following reasons^3^^,^^5^:

• Surgically sewn arch anastomosis abolishes risk of type 1A endoleaks.
• Robust arch collar, FET seal, and gelatin-treated Dacron graft enable excellent hemostasis.
• Short delivery system is easy to use.
• Ringed stent design will accommodate the curvature of the aortic arch well.
• Lower radial force may be advantageous to decrease the risk of stent-induced new entry.

Despite many favorable characteristics, 1 of the drawbacks of FET grafts is increased risk of paraplegia.^9^ Existing literature reports that paraplegia occurs in as much as 10%.^10^ In our series, we started implanting the 150-mm length more commonly but converted to 100-mm length with time, such that 64% of patients received a 100-mm-long graft, with only 1 patient (2%) experiencing permanent SCI. Another consideration is the technical difficulty of reconstructing the left subclavian artery directly. The subclavian limb of the Thoraflex graft is designed close to the arch collar, which makes direct anastomosis to the left subclavian artery challenging. Therefore, we prefer elective patients to undergo a left carotid–subclavian bypass before the FET procedure to facilitate ease of subclavian reconstruction and to minimize circulatory arrest time. This strategy also facilitates a more proximal zone 2 arch anastomosis and decreases the risk of recurrent laryngeal nerve injury. Last, we believe the ringed stent design of the Thoraflex graft is associated with less radial force than more traditional Z-stent–designed FET, which may be more advantageous in patients with acute dissection but requires careful assessment of the adequacy of stent graft deployment and expansion by transesophageal echocardiography or fluoroscopy.

The main limitation of this study is its retrospective nature and small study population. Therefore, the study needs more power for comparison of aortic pathologic processes. Also, the incidence of each complication was relatively low, and subanalyses could not be performed. Larger multicenter, prospective evidence is required.

In conclusion, the midterm outcomes of the FET procedure using the Thoraflex graft demonstrated a low mortality and an acceptable distal reintervention rate. However, close follow-up of the remnant aneurysm and timely secondary intervention are mandatory. Further experience and longer follow-up of this device will help establish its role in hybrid aortic arch repair.
