# Supplementary material for: Five-Year Outcomes of Hybrid Arch Frozen Elephant Trunk Repair With Novel Multibranched Hybrid Graft
Source: Ann Thorac Surg Short Rep. 2023 Jul 14;1(4):599–603. doi: 10.1016/j.atssr.2023.06.009 (PMC11708655; doi:10.1016/j.atssr.2023.06.009)
Supplement: Supplementary Table [file mmc1.docx]

| Table 1. Baseline characteristics | All patients (N = 50) |
| --- | --- |
| Age | 63 ± 15 |
| Female | 17(34) |
| BMI | 28 ± 6 |
| NYHA classification |  |
| I | 29(58) |
| II | 13(26) |
| III | 6(12) |
| IV | 3(6) |
| Maximum aortic diameter, mm | 61 ± 15 |
| comorbidities |  |
| Bicuspid aortic valve | 6(12) |
| Cerebrovascular disease | 13(26) |
| Connective tissue disorder | 6(12) |
| Coronary artery disease | 9(18) |
| Chronic obstructive disease | 13(26) |
| Heart failure | 8(16) |
| Diabetes mellitus | 5(10) |
| Dyslipidemia | 31(62) |
| History of tobacco use | 16(32) |
| Hypertension | 48(96) |
| Atrial fibrillation | 10(20) |
| Recent myocardial infarction | 7(14) |
| Peripheral vascular disease | 3(6) |
| Previous sternotomy | 13(26) |
| Renal failure | 1(2) |
| Aortic pathology |  |
| Aneurysm | 48(96) |
| Acute dissection | 10(20) |
| Chronic dissection | 20(40) |
| Rupture | 4(8) |

Values are mean ± standard deviation or n (%).

BMI = body mass index; NYHA = New York Hear Association
